# Advances in hepatic arterial perfusion chemotherapy for hepatic metastases

**DOI:** 10.3389/fonc.2025.1544061

**Published:** 2025-06-05

**Authors:** Haodi Ou, Xiaodong Li, Shulin Chang, Guijie Li, Feng Chen, Song Jiang

**Affiliations:** ^1^ Department of Radiology, The First Affiliated Hospital of Shandong First Medical University & Shandong Provincial Qianfoshan Hospital, Shandong Medicine and Health Key Laboratory of Abdominal Medicine Imaging, Jinan, China; ^2^ Graduate school, Shandong First Medical University & Shandong Academy of Medical Sciences, Jinan, China

**Keywords:** HAIC, hepatic metastases, treatment, chemotherapy, locoregional therapy

## Abstract

Hepatic arterial infusion chemotherapy (HAIC) is one of the local treatment modalities employed for liver metastases. HAIC targets the delivery of chemotherapy drugs to the affected area by inserting a catheter into the tumor’s blood-supplying artery. This approach not only enhances drug concentration within the tumor site, but also significantly reduces systemic side effects. Currently, there are various chemotherapeutic regimens available for HAIC in liver metastases; however, determining the optimal therapeutic agent remains elusive. This article provides a comprehensive review of HAIC dosing regimens and multimodal therapy for liver metastases originating from colorectal, breast, and gastric cancers. Meanwhile, this paper briefly outlines ongoing research on HAIC treatment for liver metastases associated with esophageal cancer, gastroenteropancreatic neuroendocrine tumors, and uveal melanoma.

## 1 Introduction

The liver serves as a preferred site for metastatic cancer due to its unique and diverse cellular and structural composition that facilitate tumor cell growth. The most frequent primary origins of liver metastases are colorectal and breast cancers. Additionally, malignancies of the digestive tract—including pancreatic, gastric, and esophageal cancers—as well as a substantial proportion of lung cancers, exhibit a high propensity for hepatic metastasis (1). The one-year survival rate for all patients with liver metastases is 15.1%, whereas it is 24.0% for patients without liver metastases (2). Therefore, the treatment of liver metastases holds particular significance.

The two main treatment modalities for liver metastases are surgical and non-surgical interventions. Complete surgical resection remains the only potentially curative treatment for liver metastases. However, the eligibility for surgical treatment is limited to less than 20% of patients because of factors such as disease burden, tumor location, hepatic functional reserve, and patient functional status (3). Consequently, non-surgical therapies have become an essential component of comprehensive treatment strategies, significantly contributing to prolonged survival and improved clinical outcomes for patients with unresectable disease. Currently, non-surgical therapies include systemic chemotherapy, local ablation therapy, radiotherapy, transcatheter arterial chemoembolization(TACE) and hepatic arterial infusion chemotherapy (HAIC). HAIC and TACE, in combination with other therapies, have been widely utilized in the management of both primary and secondary liver cancer. Currently, TACE has emerged as the first-line treatment for advanced HCC and is regarded as the optimal therapeutic choice for unresectable primary liver cancer. In cases of secondary liver cancer (i.e., metastatic liver tumors), both HAIC and TACE remain viable options when surgical resection is not feasible (4). Specifically, TACE is preferred for hypervascular hepatic metastases with small tumor burdens, whereas HAIC is more suitable for patients with large tumors, portal vein tumor thrombosis (PVTT), or those who exhibit resistance to TACE. We performed a systematic literature search in PubMed to synthesize recent advancements in HAIC therapeutic regimen and their combination therapies for hepatic metastases through 2025.

HAIC is a local treatment for liver tumors by reducing the tumor load to render it resectable (2), or by decreasing tumor size and number to achieve downstaging in advanced disease with the aim of prolonging the survival time of patients. The selection of HAIC treatment options varies depending on the origin site of the liver metastases and individual patient conditions. This review aims to provide a comprehensive overview of HAIC dosing regimens, combination regimens, and complications associated with several common types of liver metastases (colorectal cancer liver metastases, breast cancer liver metastases, and gastric cancer liver metastases). Additionally, the current research status regarding HAIC for esophageal cancer treatment, gastroenteropancreatic neuroendocrine tumors, and hepatic metastases from uveal melanoma was also presented.

## 2 Technical principles, limitations and development of HAIC

**Figure 1:**
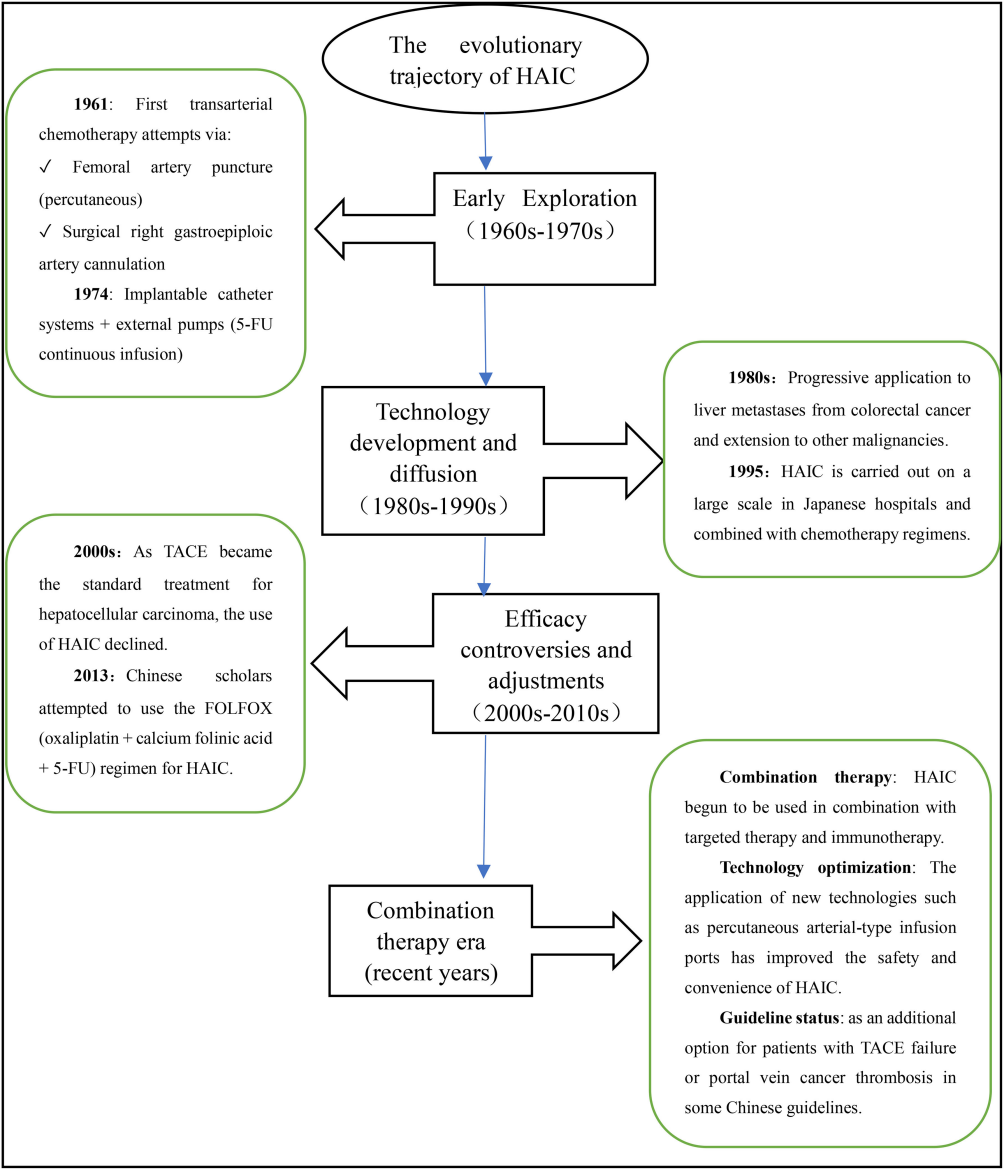
The evolutionary trajectory of HAIC.

The concept of HAIC was initially proposed by a Japanese professor in 1961 (5). In recent years, experts worldwide have increasingly focused on HAIC and made various attempts to improve its therapeutic efficacy (6). The evolutionary trajectory of HAIC therapy is illustrated in **Figure 1**.The principle underlying HAIC treatment lies in the unique dual blood supply of the liver, with 20-30% originating from the hepatic arterial system and 70-80% from the portal venous system. While normal liver tissue mainly relies on the portal vein system for blood supply, primary malignant tumors and metastases with abundant vascularization are predominantly supplied by the hepatic artery. By directly administering chemotherapy drugs into the hepatic artery, HAIC capitalizes on this characteristic blood supply pattern of liver malignancies, enabling more targeted action against tumor cells in the liver. On one hand, it reduces adverse reactions associated with systemic chemotherapy; on the other hand, it increases the total dose of chemotherapeutic drugs in the tumor region, prolongs their duration of action, improves local drug concentration within tumors, and enhances therapeutic efficacy specifically against liver metastases. This approach is particularly suitable for patients with liver metastases who are ineligible for surgical resection. At the same time, these advantages distinguish HAIC from systemic chemotherapy and have demonstrated favorable efficacy in treating liver metastatic cancer (7).

Hepatic arterial infusion chemotherapy (HAIC) is predominantly performed via percutaneous femoral artery access, where a catheter is meticulously navigated and selectively positioned within the tumor’s primary feeding vessel - most frequently the proper hepatic artery or its segmental branches - to achieve optimal regional chemotherapy delivery. This technique remains effective for bilobar metastatic disease through strategic catheter placement in the proper hepatic artery. When anatomical variations are present, preoperative hepatic arteriography enables precise identification of tumor-specific vascular supply, guiding accurate catheter positioning within the dominant feeding arteries. In cases of complex vascular anatomy with multiple feeding vessels, the catheter is optimally deployed in the principal supplying artery while supplementary embolization of accessory vessels can be performed, thereby enhancing therapeutic efficacy by concentrating chemotherapeutic agents within the primary tumor vasculature while simultaneously reducing non-target tissue exposure. In cases of hepatic arterial anatomical variations, requirements for prolonged therapeutic intervention, or unsuccessful percutaneous attempts, surgical catheter placement may be alternatively pursued. The procedure involves either an open or laparoscopic surgical approach for precise catheter placement - the distal tip is positioned within the target artery and surgically secured, while the proximal end is connected to a subcutaneous infusion port to enable sustained therapeutic delivery. This administration can be achieved through continuous perfusion or cyclical treatment modalities. In addition, vascular embolic agents are combined to reduce the rate of hepatic arterial blood flow and prolong the residence time of drugs within the lesion in order to enhance local drug concentration. Many studies have shown that HAIC exhibits superior survival rates and efficiency compared to systemic chemotherapy (7).

HAIC also carries potential risks and side effects, such as hepatic aneurysm and cholangitis caused by surgical operation errors. Several studies have shown that while HAIC can extend the survival of patients with intrahepatic cholangiocarcinoma, it is also associated with a higher incidence of toxicity (7). It is important to note that patients undergoing HAIC treatment may also experience hepatic artery occlusion (HAO), which necessitates discontinuation of HAIC therapy and transition to systemic chemotherapy. However, in cases of hepatic artery pseudo-occlusion, only the catheterization system needs to be replaced without requiring any alteration in the treatment regimen. Yamaura H et al. reported two cases where HAO was diagnosed through arteriography but the hepatic artery was actually patent; thus, recognizing pseudo-occlusion of the hepatic artery is crucial for subsequent treatment (8).

## 3 HAIC in colorectal cancer liver metastases

Colorectal cancer is ranked as the fourth most prevalent malignant tumor globally (9). Liver metastasis, predominantly occurring in colorectal cancer patients, stands as the leading cause of mortality (7). Surgical intervention combined with systemic chemotherapy represents the most effective treatment for patients with colorectal cancer liver metastases (CLM). Nevertheless, not all patients are suitable candidates for surgical resection and even among those who undergo surgery, over 60% experience recurrence of liver tumor following partial hepatectomy. Therefore, we aimed to investigate whether HAIC could be utilized as a potential therapeutic approach for treating CLM (10). In managing CLM, HAIC has been employed either as salvage therapy for advanced liver metastases or as adjuvant therapy subsequent to hepatic resection (7). This review further explores the benefits of using HAIC in patients with unresectable CLM.

### 3.1 5-Fluorouracil in HAIC for colorectal cancer liver metastases

The application of 5-FU in HAIC can safely and effectively improve liver function and provide surgical opportunities or improve prognosis for patients with liver metastases of colorectal cancer (5). As shown in **Table 1**, HAIC, either alone or in combination with other agents, is a treatment option for patients with colorectal cancer liver metastases who have failed standard systemic chemotherapy. Nishiofuku H et al. conducted a study on 55 patients who experienced disease progression during standard systemic chemotherapy with oxaliplatin, irinotecan, and 5-FU. After receiving HAIC treatment with 5-FU, the overall response rate was found to be 18.2% and the disease control rate was 70.9%. The median progression-free survival and median overall survival (MOS) were reported as 2.8 months and 6.7 months, respectively (9). Similarly, in another study involving 137 patients with liver metastases from colorectal cancer who exhibited poor results with systemic chemotherapy conducted by Sato Y et al., it was observed that the combination of HAIC and 5-FU demonstrated therapeutic efficacy while reducing the incidence of serious adverse events compared to standard systemic chemotherapy (11).

**Table 1:** HAIC for colorectal cancer liver metastases.

Author	Year	Numbers	HAIC regimen	OS (month)	MST (month)	PFS (month)	ORR	DCR
Nishiofuku H (9)	2010	55	5-FU	6.7	–	2.8	18.2%	70.9%
Sato Y (11)	2020	137	5-FU	4.8	–	–	12.4%	64%
Goi T (12)	2015	10	5-FU, LV	9	–	–	–	70%
Guo J-H (13)	2017	24	5-FU, oxaliplatin vs oxaliplatin,raltitrexed	–	15.4 vs 20.6	6.6 vs 4.9	–	–

Missing data are uniformly marked as “-” (not reported in the literature).

The combination of 5-FU with other drugs in HAIC is frequently used to treat patients with liver metastases from colorectal cancer. A study by Goi T et al. demonstrated the effectiveness of combining 5-FU with calcium folinate in HAIC for treating CLM resistant to systemic chemotherapy or unresponsive to molecularly targeted therapies. The study reported a disease control rate of 70% and a median survival time of 9 months, ranging from 2 to 16 months. This drug combination not only improves patients’ quality of life, but also induces apoptosis or death of drug-resistant colorectal cancer cells due to higher drug concentration (12). Furthermore, HAIC application with a combination of 5-FU and oxaliplatin is an effective and safe alternative to raltitrexed and oxaliplatin. Guo J-H et al.’s research showed no significant difference in overall survival (OS) between the two combinations, except for differences in side effects; leukopenia was more common in the 5-FU group while liver disease was more common in the raltitrexed group (13).

In addition, HAIC combined with systemic therapy has been proven feasible, Zhang Y et al. mentioned a triple regimen consisting of oxaliplatin, irinotecan, and concurrent administration of systemic cetuximab along with 5-FU for treating 64 patients with CRC. The outcomes revealed a resection rate ranging from R0 to R1 at 29.7%, a remission rate of 40.6%, and a median progression-free survival period of 9.3 months (11). Furthermore, a case-control study revealed significantly improved survival outcomes in patients with colorectal liver metastases (CRLM) treated with 5-FU-based HAIC plus systemic chemotherapy versus systemic chemotherapy alone, with median overall survival (OS) of 32.8 months and 15.3 months, respectively (p<0.001). This represents a clinically meaningful survival advantage of 17.5 months (14).These findings indicate that combining HAIC with systemic chemotherapy is emerging as a promising therapeutic strategy for CRLM. However, current evidence remains limited by the preliminary status of ongoing large prospective trials evaluating this combined approach. Consequently, well-designed studies are still needed to establish standardized protocols for HAIC plus systemic therapy in CRLM management (4).

Other researchers have also explored the combination therapy approach that involves combining HAIC (5-FU) with radiofrequency ablation (RFA) to manage patients with unresectable CLM, observing stable or complete or partial remissions among individuals who underwent this treatment course. Notably, HAIC followed by RFA resulted in complete necrosis of the residual lesions, thus providing an alternative therapeutic option for patients facing unresectable CLM (15).

The administration of HAIC combined with 5-FU has been associated with the occurrence of gastric injury, hyperbilirubinemia, hepatic abscesses, and leukopenia in certain patients. It is worth noting that the gastric injury observed in HAIC resembles reactive gastritis induced by chemical irritants; some patients present with gastric ulcers while others exhibit glandular isoforms induced by 5-FU treatment. However, it should be emphasized that glandular anisotropy is a specific characteristic of HAIC and cannot be interpreted as carcinoma despite morphological concerns (16).

### 3.2 Floxuridine in HAIC for colorectal cancer liver metastases

It has been shown that the preoperative use of HAIC with FUDR in patients with advanced liver disease is associated with a reduced rate of hepatic recurrence, improved long-term survival, and no negative impact on post-hepatectomy prognosis. The study conducted by Pulitanò C et al. involved 50 patients with CLM who were divided into two groups: one group received preoperative HAIC, while the other group did not receive HAIC prior to surgery. Subsequently, all patients underwent hepatectomy. The final results showed that the HAIC group exhibited higher overall disease-free survival rates at 1 and 3 years post-hepatectomy, as well as improved OS rates at 1, 3, and 5 years after CLM diagnosis compared to the non-HAIC treated group (17). These findings further support the feasibility of performing hepatectomy following preoperative application of FUDR for HAIC.

The combination of HAIC (FUDR) with systemic chemotherapy has been shown to effectively manage liver disease, prolong treatment duration, and improve resectability (18). A small randomized clinical trial conducted in Italy demonstrated that hepatic artery infusion of both FUDR and high-dose systemic FUDR was superior to hepatic artery infusion of FUDR alone in patients with initially unresectable liver metastases from colorectal cancer (median survival: 20 months vs. 14 months). This finding confirms the potential benefits of combining HAIC (FUDR) with systemic chemotherapy (18). Buisman F E et al. also pointed out the promising role of adjuvant HAIC, especially when accompanied by FUDR and systemic therapy, as a treatment option for patients with resectable CLM (19). For patients with resectable colorectal liver metastases (CLM), adjuvant HAIC - particularly with floxuridine (FUDR) combined with systemic chemotherapy - represents a promising therapeutic strategy. In a clinical study of 49 CRLM patients receiving HAIC with fluorouracil plus systemic oxaliplatin/irinotecan combination therapy, exceptional outcomes were achieved: a 92% overall response rate (ORR) and a median overall survival (OS) of 39.8 months (4).

FUDR exhibits hepatotoxicity, characterized by mechanisms such as hepatocellular necrosis, steatosis, cholestasis, central venous sclerosis, and portal triad. The specific clinical manifestations may vary depending on the dose and duration of HAIC (20). Doria M I et al. found that all patients with CLM developed hepatitis; however, they responded positively to HAIC (FUDR) treatment (20). Anderson S D et al. discovered that jaundice is a common occurrence in many patients with CLM undergoing HAIC (FUDR). This condition is attributed to chemical hepatitis and bile duct stenosis, as well as complications associated with arterial chemotherapy rather than tumor progression obstruction (21).

### 3.3 Oxaliplatin in HAIC for colorectal cancer liver metastases

Oxaliplatin, a third-generation platinum compound, serves as the cornerstone of first-line chemotherapy for colorectal cancer (CRC). Despite its clinical efficacy, dose-limiting oxaliplatin-induced peripheral neuropathy (OIPN) frequently necessitates treatment discontinuation. To circumvent this limitation, we implemented hepatic arterial infusion chemotherapy (HAIC) as a targeted delivery approach for oxaliplatin administration (22).

Oxaliplatin-based adjuvant HAIC demonstrates clinical feasibility. The study shows that the combination of oxaliplatin-based HAIC with 5-FU/FUDR demonstrates robust efficacy in refractory colorectal cancer liver metastases (CRLM). In this prospective trial involving 21 patients with disease progression after standard systemic chemotherapy, the regimen achieved an objective response rate (ORR) of 28.6% and a disease control rate (DCR) of 95.2%. Notably, 6 patients (28.6%) attained partial remission, while 7 patients (33.3%) subsequently qualified for conversion surgery, underscoring the potential of this approach to enable curative-intent interventions in initially unresectable cases (23).

Clinical evidence demonstrates that the combination of oxaliplatin-based HAIC with systemic chemotherapy and molecular targeted agents represents a therapeutically viable and well-tolerated approach for patients with initially unresectable colorectal liver metastases (CRLM) (24). A phase II clinical trial evaluating first-line treatment with oxaliplatin-based hepatic arterial infusion chemotherapy (HAIC) combined with systemic 5-fluorouracil and cetuximab demonstrated remarkable efficacy in 35 patients with colorectal liver metastases (CRLM). The regimen achieved an outstanding objective response rate (ORR) of 88%, with median progression-free survival (PFS) and overall survival (OS) reaching 17.9 months and 46.3 months (25).

### 3.4 Prognosis and postoperative response to HAIC for colorectal cancer liver metastases

The prognosis of patients with CLM is affected not only by primary tumor surgery but also by the treatment for liver metastases. In cases where surgical removal of the liver metastases is not feasible, we consider treating patients with HAIC (15). Therefore, accurately predicting the efficacy and prognosis of HAIC treatment becomes particularly important (26).When evaluating the effectiveness of HAIC, computed tomography (CT)-based criteria according to RECIST guidelines are widely used as a standard for assessing solid tumors. Liu P et al. conducted a study where they extracted the imaging features from CT scans taken before and after HAIC treatment and developed a model to predict the OS. Their findings indicate that analyzing preprocessed CT images can provide early prognostic information for patients with advanced unresectable colorectal cancer undergoing HAIC treatment. Moreover, this method demonstrates high accuracy and feasibility (26).

In addition, the relative change in the minimal ADC value (%minADC) on DWI may be a valuable tool for early detection of response to 5-FU combined with HAIC in patients with colorectal liver metastases (26).Marugami N et al. conducted pre- and post-HAIC DWI scans on patients with CLM, dividing them into responding and non-responding groups according to the changes in liver metastases. The results revealed significantly higher values of the minimum apparent diffusion coefficient (%minADC) and the mean apparent diffusion coefficient (%meanADC) in the responding group compared to the non-responding group. Furthermore, %minADC (100% sensitivity and 92.6% specificity) exhibited higher diagnostic accuracy than %meanADC (66.7% sensitivity and 74.1% specificity). These findings highlight the potential value of assessing relative changes in minimum ADC values on DWI for guiding HAIC treatment decisions (27).

## 4 HAIC in breast cancer liver metastases

Breast cancer is one of the most prevalent malignancies in women, and liver metastasis represents a common occurrence. The primary objectives for treating breast cancer liver metastases are to alleviate symptoms, control disease progression, extend survival rates, and improve quality of life. Hepatectomy for patients with liver metastases from breast cancer necessitates ensuring three essential factors: (1) low surgical risk; (2) complete resectability; and (3) absence of extrahepatic disease (except for rare bone metastases) (28). Due to the limited number of patients who meet these criteria, treatment options for breast cancer liver metastases typically involve a combination of therapeutic regimens, including systemic chemotherapy, surgical resection, RFA, radiotherapy, or HAIC. Therefore, HAIC can be considered in cases where surgical resection or RFA is not feasible and conventional systemic chemotherapy has proven ineffective against breast cancer liver metastases (29). Various HAIC regimens catering to patients from different backgrounds have been reported in the literature, but the efficacy of HAIC may vary across different patient populations. Below we discuss the various treatment options for HAIC, some of which can be seen in **Table 2**.

**Table 2:** HAIC for breast cancer liver metastases.

Author	Year	numbers	HAIC regimen	OS (month)	MST (month)	PFS (month)	ORR	DCR
Masuda T (29)	2021	1	5-FU, epirubicin	15	–	–	–	–
Tewes M (35)	2017	70	5-FU, mitomycin, melphalan	7	–	2	–	–
Furuta M (36)	2020	57	5-FU, epirubicin, mitomycin-C (FEM)	11.3	–	–	63%	–
Hsiao J-H (38)	2018	42	mitoxantrone, 5-fluorouracil, Folinic, acid, cisplatin	19.3	–	8.4	48%	67%
Shi H- B (42)	2021	19	gemcitabine, FUDR	13.1	–	–	73.7%	–

Missing data are uniformly marked as “-” (not reported in the literature).

HAIC can be used as a surgical alternative for patients with liver metastases from breast cancer who are ineligible for surgical resection. Fujito T et al. reported a case of multiple liver metastases from breast cancer in a 46-year-old man. In this patient, the size of the liver metastases gradually decreased and tumor markers returned to the normal range after HAIC and systemic chemoendocrine therapy. Subsequent hepatectomy confirmed the disappearance of cancer cells (30). Multimodal therapies, including HAIC, are also suitable for patients with liver metastases from breast cancer, such as HAIC combined with cytotoxic chemotherapy and endocrine ablation. Peetz M et al. found that patients with liver metastases from breast cancer treated with cytotoxic chemotherapy had a longer mean survival time when they received a combination of hepatic arterial perfusion chemotherapy and hormone ablation procedures compared to those who only received hormone ablation therapy. Moreover, both groups had longer mean survival time than expected (three to nine months), indicating that multimodal therapy, including HAIC, is an effective treatment option for patients with liver metastases from breast cancer (31).

It is important to note that while HAIC has demonstrated efficacy in treating liver metastases from breast cancer, the response to treatment varies depending on the morphologic type of liver metastasis. Liver metastases can be categorized into four types based on their morphology: isolated mass type, multiple nodular type, diffuse small nodular type, and mixed type. By examining the outcomes of arterial infusion chemotherapy in 21 patients with hepatic metastases from breast cancer, Kakuta T et al. found that cases of isolated mass type, multiple nodule type, and mixed type showed a partial response to treatment, whereas cases of diffuse small nodule type exhibited no change or progression of the lesion. This indicates a correlation between the morphologic subtype of the hepatic metastasis and the response to HAIC treatment (32). Furthermore, Y M et al., when evaluating the outcomes of breast cancer patients treated with HAIC after surgery, observed shrinkage of metastatic lesions in 4 cases (33.3%), accompanied by irregularities in the hepatic surface margins in another 3 cases (25.0%). They also noted a high percentage of extensive compensatory hepatic malformations in 5 other cases (41.7%). These changes were more pronounced with an increased number of liver metastases and were frequently associated with hepatic degeneration; thus, caution must be exercised when assessing the efficacy of treatment (33).

### 4.1 5-FU in HAIC for the treatment of liver metastases from breast cancer

The optimal dosing regimen for HAIC in the treatment of patients with liver metastases from breast cancer has not been determined (34). Nonetheless, according to pharmacokinetic studies, 5-FU is considered one of the preferred drugs for local hepatic perfusion (34). It has a high hepatic extraction rate, which not only improves the therapeutic efficacy but also minimizes systemic toxicity (34). In the following discussion, we explore the therapeutic options related to the application of 5-FU in HAIC.

The combination of 5-FU and mitomycin is a safe and effective palliative treatment option for breast cancer patients with extensive liver metastases after long-term treatment. Tewes M et al., in their study involving the HAIC regimen (mitomycin, melphalan, 5-FU) on 70 such patients, demonstrated that HAIC (especially the combined use of mitomycin and 5-FU) represents a viable therapeutic option. Additionally, liver metastasis scoring can be used to closely monitor and mitigate treatment toxicity, thereby enabling patients to achieve more enduring benefits (35). For advanced breast cancer patients ineligible for systemic drug therapy due to liver metastases, the application of HAIC with 5-FU and epothilone may serve as an alternative option. Masuda T et al. reported a case study involving a 64-year-old woman who had undergone breast cancer surgery but developed multiple liver metastases. Despite declining systemic medications for further treatment, she opted for HAIC with 5-FU and epothilone while receiving paclitaxel therapy for over one year. CT findings revealed partial hepatic response along with decreased levels of CEA and CA15–3 markers; moreover, she survived for one year and three months after undergoing HAIC treatment. These results indicate that combining 5-FU with epothilone is a favorable therapeutic choice for breast cancer patients refusing systemic therapy in cases of multiple liver metastases (29). The FEM regimen consisting of 5-FU, epothilone, and mitomycin-C has demonstrated efficacy in the treatment of breast cancer with advanced liver metastases. Furuta M et al. conducted a study involving 57 patients with liver metastases from breast cancer who were unresponsive to standard systemic chemotherapy. These patients received HAIC using 5-FU, epothilone, and mitomycin C ((FEM) regimen), resulting in a MOS of 11.3 months and an objective effectiveness rate of 63% (36). The effect of combining this regimen with systemic endocrine therapy was explored by Onogawa S et al., who reported a case study involving a patient with breast cancer that developed liver metastases 14 years after radical mastectomy. The combination treatment approach utilizing HAIC (FEM regimen) along with systemic endocrine therapy (medroxyprogesterone acetate and fadazole hydrochloride hydrate) successfully controlled the growth of the liver tumor. This suggests that combining HAIC (FEM regimen) with systemic endocrine therapy can be considered as a therapeutic option for patients with extensive liver metastases (37). In addition, Hsiao J-H et al. investigated the use of HAIC in patients with progressive liver disease by employing a combination treatment approach involving 5-FU, mitoxantrone, folinic acid, and cisplatin. Their study on 42 breast cancer patients who developed progressive liver metastases following systemic therapy showed favorable outcomes achieved through HAIC treatment (38).

### 4.2 Docetaxel in HAIC for the treatment of liver metastases from breast cancer

Docetaxel is commonly used in the treatment of breast cancer, especially for patients with advanced or metastatic breast cancer. The specific treatment regimen for each patient needs to be tailored according to their individual circumstances (e.g., the molecular subtype of breast cancer, the size and number of liver metastases, etc.). The clinician should also consider the dosage and frequency of docetaxel administration, as well as its combination with other medications. Kim S J et al. reported a case study on a patient with liver metastases from breast cancer who underwent radical mastectomy but showed no improvement in liver metastases after CEF treatment (cyclophosphamide, epirubicin, and 5-FU). However, the hepatic metastases were reduced after hepatic arterial infusion of docetaxel, with only mild side effects including leukopenia, generalized fatigue, and fever. The application of HAIC docetaxel not only involved a lower dosage compared to systemic administration but also resulted in a favorable response and minimal side effects (39).

Hepatic arterial infusion of docetaxel, in combination with systemic administration of albumin-bound paclitaxel, represents a reliable treatment for liver metastases originating from breast cancer. Satoh E et al. reported a case of a 50-year-old woman with liver metastases from left breast cancer who exhibited reduction in the size of a solitary liver metastasis following postoperative hepatic arterial infusion of docetaxel. Subsequently, the patient achieved partial remission (PR) after 7 years when multiple liver and lung metastases were treated with systemic administration of albumin combined with paclitaxel chemotherapy (40). Additionally, it has been shown that combining hepatic arterial infusion of docetaxel with systemic trastuzumab chemotherapy may hold significance in the treatment of breast cancer patients with liver metastases. Hashimoto K et al. reported a case of a 43-year-old woman with multiple liver metastases from right breast cancer (pT3N3aM1, stage IV, ER (-), PgR (-), and HER2 (3+)), who achieved complete disappearance of the liver metastases and reduction in tumor markers to the normal range on CT after receiving postoperative treatment consisting of hepatic arterial perfusion of docetaxel combined with systemic trastuzumab. Notably, the only observed side effects were grade 1 nausea, indicating that this combination chemotherapy regimen is not only safe but also effective for patients with liver metastases (41).

Nonetheless, the potential adverse effects of docetaxel, such as myelosuppression, alopecia, emesis, among others, should be taken into consideration. Close monitoring and prompt reporting of any discomfort experienced by patients during treatment is essential.

### 4.3 Other drugs in HAIC for breast cancer liver metastases

FUDR and gemcitabine are commonly utilized in HAIC for the treatment of liver metastases from breast cancer. These two agents have different roles and mechanisms in local chemotherapy, and their combined application, either with systemic chemotherapy or alone, has demonstrated efficacy in achieving high rates of local response and prolonged MOS in patients with liver metastases from breast cancer, while maintaining relatively low toxicity levels. Shi H-B et al. conducted a study involving 19 patients with hepatic metastases from breast cancer who received systemic chemotherapy along with HAIC using gemcitabine combined with FUDR. The treatment resulted in an overall response rate (ORR) of 73.7% for intrahepatic lesions and a MOS of 13.1 months. Grade 3 adverse effects observed included leukopenia, neutropenia, and diarrhea (42).

Gemcitabine, a widely used chemotherapeutic agent, exerts its antiproliferative effects on cancer cells by impeding DNA synthesis. Mitsuyama S et al. reported a case of liver metastases in a 51-year-old woman who developed the condition one year after undergoing postoperative chemotherapy and radiotherapy for right-sided breast cancer. Despite failed attempts with multiple therapies, the patient demonstrated a positive response to all metastatic lesions following 3 cycles of HAIC combined with gemcitabine. These findings provide evidence supporting the safety and efficacy of gemcitabine monotherapy for managing liver metastases from breast cancer (43).

Paclitaxel is extensively utilized as a chemotherapy medication known for its remarkable therapeutic efficacy against various types of cancers, including breast cancer. When dealing with liver metastases from breast cancer, it is possible to administer paclitaxel via HAIC. A study conducted by Iwamoto S et al. documented a successful case where hepatic arterial infusion therapy using paclitaxel resulted in complete disappearance of liver metastasis three years post-mastectomy and adjuvant chemotherapy. The observed side effects were limited to grade 2 alopecia and grade 1 peripheral neuropathy (44).This indicates that utilizing paclitaxel in HAIC for treating liver metastases arising from breast cancer could be considered; however, individualized decision-making should rely upon careful evaluation by medical professionals considering each patient’s unique circumstances.

## 5 HAIC in liver metastasis of gastric cancer

Gastric cancer (GC) ranks as the fifth most frequently diagnosed malignancy and the third leading cause of cancer-related mortality globally. The most common metastatic sites of GC include the liver, peritoneum, and bone. Data from the Surveillance, Epidemiology, and End Results (SEER) database indicate that approximately 34% of GC patients present with distant metastases at diagnosis, with hepatic metastases accounting for 4–14% of initial clinical manifestations in this population (45). Encouraged by favorable outcomes observed in CLM, there has been an increased focus on curative or palliative treatment options for liver metastases of gastric cancer. Current clinical modalities employed for managing liver metastases from gastric cancer encompass hepatectomy, systemic chemotherapy, RFA, HAIC, and palliative gastrectomy (46). While systemic chemotherapy is recognized as the standard therapy for treating liver metastases of gastric cancer, its survival benefits remain unsatisfactory. As shown in **Table 3**, HAIC is expected to be a potential treatment for liver metastases in gastric cancer due to its significant local efficacy (47). Moreover, combining HAIC with systemic chemotherapy may further enhance the prognosis improvement, as reported by Takeno A et al., who studied 19 patients treated with HAIC. The findings demonstrated a remission rate of 26% (CR 3, PR 2, PD 14) and a median survival time of 11.9 months after the diagnosis of liver metastases (48). Combining HAIC therapy with systemic chemotherapy and/or surgical intervention holds great promise as a potential therapeutic approach for patients with gastric cancer-related liver metastases.

**Table 3:** HAIC for gastric cancer liver metastases.

Author	Year	numbers	HAIC regimen	OS (month)	MST (month)	PFS (month)	ORR	DCR
Takeno A (48)	2003	19	–	–	11.9	–	26%	–
Ojima H (49)	2007	37	5-FU	–	19.2	–	83%	–
Kumada T (50)	1999	1	5-fluorouracil, epirubicin, mitomycin-C	–	10.5	–	55.6%	–
Seki H (58)	2016	14	5-fluorouracil, epirubicin, mitomycin-C	–	12.7	–	42.9%	–
Katayanagi S (59)	1999	10	5-fluorouracil, epirubicin, mitomycin-C	–	25.2	–	–	–

Missing data are uniformly marked as “-” (not reported in the literature).

### 5.1 5-FU in HAIC for liver metastases from gastric cancer

There are various chemotherapeutic regimens available for HAIC, with 5-FU commonly used for the treatment of liver metastases from gastric cancer (49). Toyokawa T et al. reported a case demonstrating complete remission following low-dose HAIC with 5-FU. In this instance, a male patient with advanced gastric cancer developed liver metastases post-surgery and achieved complete clinical remission after 8 months of systemic chemotherapy and HAIC treatment (using 5-FU). No serious adverse effects were observed, and the patient remained disease-free for more than 12 years. While HAIC can be considered as a potential treatment option for patients with resectable liver metastases from gastric cancer, it is important to note that only one case was included in this study; thus further research is required to validate the effectiveness of HAIC in such patients (50). Ojima H et al. investigated the outcomes of HAIC treatment in 37 patients presenting synchronous multiple liver metastases from gastric cancer, reporting an impressive HAIC remission rate of 83% when using 5-FU. These findings highlight the effectiveness of HAIC as a method for controlling liver metastasis. Nevertheless, it should be noted that survival rates were not improved by HAIC treatment alone; gastrectomy may play a crucial role in improving the OS (49).

Multimodality therapy may also be considered as a therapeutic option for patients with liver metastases from gastric cancer. The combination of HAIC using 5-FU and transcatheter arterial chemoembolization (TACE) has shown potential efficacy in treating highly vascular liver metastases caused by gastric cancer. For example, Tanigawa T et al. reported an 83-year-old patient who had undergone surgery for gastric cancer but showed no significant response to systemic chemotherapy. This patient received TACE with degradable starch microspheres (DSM) plus mitomycin C, followed by HAIC with high-dose 5-FU and adjuvant chemotherapy with paclitaxel. Remarkably, this patient remained recurrence-free at the 8-month follow-up (51). Additionally, HAIC with 5-FU can be combined with RFA or hepatectomy to enhance treatment outcomes. For example, Hasuike Y et al. reported four cases of simple liver metastases after gastric cancer resection in which patients underwent hepatic resection or RFA after hepatic arterial perfusion with 5-FU and subsequent HAIC administration. Two patients survived without recurrence for durations of 12 months and 21 months, respectively. One patient with prostate cancer survived for 22 months without recurrence; while the last patient experienced recurrence at a non-hepatic artery supplied site after surviving for 36 months (52). These findings demonstrate the effectiveness of combining HAIC with hepatectomy and RFA in treating solitary liver metastasis from gastric cancer.

### 5.2 5-FU and cisplatin in HAIC for liver metastases from gastric cancer

Gastric cancers that produce alpha-fetoprotein (AFP) are highly prone to liver and lymph node metastases, resulting in a poor prognosis (53). However, HAIC with 5-FU and cisplatin has shown promise as an effective and valuable treatment modality for AFP-producing gastric cancer presenting only with liver metastases after radical gastrectomy (54). Moritani M et al. reported a case study where HAIC was administered once a month, consisting of low-dose CDDP and intermittent administration of 5-FU. This resulted in normalized AFP levels without recurrence for 1 year and 11 months post-treatment (55).This treatment regimen can also be combined with systemic chemotherapy to manage AFP-producing gastric cancers. Doi Y et al. presented a case study involving a patient with multiple liver metastases from gastric cancer exhibiting significantly elevated AFP levels (588.9 ng/mL). The patient received HAIC (5-FU, cisplatin) along with systemic chemotherapy (paclitaxel/remoximab (PTX/RAM)), leading to decreased AFP levels and complete remission as confirmed by CT scan findings. Based on experience, it is suggested that combining HAIC (5-FU/CDDP) with systemic chemotherapy including the RAM regimen could be an effective approach for treating liver metastases arising from methemoglobin-producing gastric cancer (53).

Gastric endocrine carcinoma (EC) exhibits markedly aggressive behavior and a dismal prognosis, with death typically occurring within one year of diagnosis. The optimal treatment for this rare gastric tumor remains undetermined. However, HAIC may represent an effective therapeutic approach for this rare gastric tumor. Nishimura M et al. reported a case of early-stage gastric EC with multiple liver metastases shortly after surgery, where in the tumor almost completely regressed following treatment with cisplatin and 5-FU. Therefore, HAIC therapy shows promise as an efficacious method for managing hepatic metastases arising from gastric EC (56).

### 5.3 5-FU, epirubicin and mitomycin C in HAIC for the treatment of liver metastases from gastric cancer

The FEM chemotherapy regimen is a combination of 5-FU, epirubicin, and mitomycin C drugs designed to enhance the therapeutic efficacy by utilizing different drug mechanisms and potentially reducing the resistance caused by single-drug treatment. To evaluate the effectiveness of the FEM regimen in HAIC application, Kumada T et al. administered this regimen (5-FU 333 mg/m(2) weekly, epirubicin 30 mg/m(2) every 4 weeks, and mitomycin C 2.7 mg/m(2) every 2 weeks) to patients with liver metastases from gastric cancer after hemihepatectomy. The remission rate after HAIC treatment was 55.6%, and the mean survival rate at 50% was 10.5 months, demonstrating that HAIC application of the FEM regimen can achieve a high remission rate in patients with liver metastases from gastric cancer (57). In addition, the FEM regimen may also be considered for HAIC treatment in patients with liver metastases from gastric cancer who have failed systemic chemotherapy. Seki H et al. conducted a review on 14 patients with hepatic metastases from gastric cancer that progressed during systemic S-1 plus cisplatin therapy and reported an objective remission rate of 42.9%, including a complete remission rate of 14.3%. The median survival after treatment with the FEM regimen was found to be 12.7 months. These findings provide evidence supporting the feasibility and effectiveness of HAIC using the FEM regimen as a second-line therapy option for achieving long-term disease control in cases of gastric cancer liver metastases (58). Additionally, combining HAIC with percutaneous ethanol injection (PEI) is also an effective method for treating such liver metastases. Katayanagi S et al. reported a median survival time of 25.2 months in 10 patients with advanced gastric cancer liver metastases who received PEI therapy in combination with HAIC using the FEM regimen. This experience suggests that combining HAIC therapy with FEM and PEI can be considered as a therapeutic option for patients with advanced gastric cancer liver metastases (59).

Gastric cancer liver metastasis (GLM) frequently recurs in the remaining liver after hepatectomy, but adjuvant HAIC therapy following hemihepatectomy for GLM can effectively prevent such recurrence. In a study by Fukami Y et al., they selected 14 patients who underwent hemihepatectomy for GLM and received the FEM regimen for 6 months. Among the eleven patients who completed HAIC therapy, the overall 5-year survival rate was found to be 43%, with recurrence observed in 8 patients. The recurrent sites included lungs, lymph nodes, peritoneal dissemination, brain, pleura, and multiple locations without any evidence of residual liver recurrence. Additionally, Kumada T et al.’s research revealed that most deaths among responders were attributed to extrahepatic lesions (57). This further supports the notion that adjunctive HAIC therapy after hemihepatectomy for GLM not only prevents residual liver recurrence but also contributes to long-term survival (60).

## 6 HAIC in liver metastases of other cancers

Esophageal cancer is a prevalent malignancy of the digestive tract, and China exhibits a high incidence rate of this disease. Progressive dysphagia represents a typical symptom associated with esophageal cancer. Liver metastasis in esophageal cancer signifies an advanced stage, often characterized by heightened severity. In their study, Dong F et al. investigated the potential therapeutic efficacy of HAIC in 33 patients with liver metastatic esophageal cancer. As shown in **Table 4**, The results revealed an ORR of 48.5%, DCR of 93.9%, PFS duration of 4.8 months, and MOS time of 6.4 months following administration of HAIC treatment to these patients. Given its demonstrated effectiveness and tolerability among individuals with liver metastases from esophageal cancer, HAIC may function as a viable regional treatment option for such cases (61).

**Table 4:** HAIC for other liver metastases.

Author	Year	numbers	HAIC regimen	OS (month)	MST (month)	PFS (month)	ORR	DCR
Dong F (61)	2023	33	5-FU, platinum, docetaxel	6.4	–	4.8	48.5%	93.9%

Missing data are uniformly marked as “-” (not reported in the literature).

Gastroenteropancreatic neuroendocrine tumors (GEP-NETs) are a diverse group of tumors that originate from peptidergic neurons and neuroendocrine cells in the gastrointestinal tract and pancreas, accounting for approximately 55% to 70% of all neuroendocrine tumors in the body. These tumors are characterized by their ability to produce certain bioactive peptides. Metastases often present at the time of diagnosis, with liver being the most common site, leading to liver failure and contributing to 80% mortality rate among patients. The key prognostic factors affecting patient survival are liver involvement and hepatic tumor burden (62). Vascular intervention through transarterial embolization (TAE) or HAIC has been proven safe and effective for patients with GEP-NET liver metastases. In a study conducted by Liu P et al., involving 50 patients with GEP-NET liver metastases who underwent TAE or HAIC, there were 4 complete remissions (CR), 28 partial remissions (PR), and 18 stable diseases (SD). This suggests that interventional therapy using TAE or HAIC is beneficial for patients predominantly affected by hepatic metastases from GEP-NETs (63).

Uveal malignant melanoma is the most common type of malignant intraocular tumors in adults, accounting for the highest incidence of intraocular tumors in foreign countries and ranking as the second most common intraocular tumor after retinoblastoma in China. This highly aggressive tumor has a tendency to metastasize through blood circulation, with liver metastases occurring in approximately 85% of cases and carrying a dismal prognosis. Patients with hepatic metastases from uveal melanoma can be treated with HAIC, which involves localized tumor control through regular and repetitive injections of melphalan or formostatin into either the hepatic artery or hepatic lobar artery (64).

However, there are limited clinical studies on HAIC for the treatment of liver metastases in three specific tumor types, namely esophageal cancer, gastrointestinal and pancreatic neuroendocrine tumors, and uveal melanoma. Therefore, it is crucial to carefully select an optimal treatment plan based on the patient’s condition.

## 7 Conclusion

The HAIC treatment is a valuable option for patients with advanced unresectable liver metastases. Commonly used drugs in the HAIC treatment of liver metastases include 5-FU, FUDR, epirubicin, cisplatin, among others. Typically, a combination of multiple chemotherapeutic agents is utilized to enhance the therapeutic efficacy through synergistic effects and reduce the resistance associated with monotherapy. Furthermore, in the management of colorectal cancer liver metastasis, breast cancer liver metastasis, and GLM, according to the specific conditions of patients, the multimodal approaches combining HAIC with surgical resection, TACE, systemic chemotherapy, molecular targeted therapy, and RFA have demonstrated prolonged survival outcomes of patients with advanced liver metastases. Additionally, HAIC may hold potential as a therapeutic strategy for treating GEP-NET liver metastases, uveal melanoma-associated hepatic involvement, and esophageal cancer-related hepatic dissemination.

However, the efficacy of HAIC is also constrained by various factors, such as the degree of cirrhosis and liver function status. Patients with impaired liver function experience heightened adverse effects and poor therapeutic effectiveness. Treatment outcomes are typically less favorable in cases involving embolism in the main trunk of the vasculature or when there are excessively large and numerous tumors. Furthermore, HAIC therapy has limitations in controlling the growth of extrahepatic tumors. Additionally, many studies have a relatively small sample size, necessitating specific discussions on optimizing treatment plans and clinical applications based on individual patient conditions.

There exists a plethora of treatment options available for liver metastases, and the selection of the most appropriate treatment plan requires careful consideration of patient-specific factors such as the size and number of metastases, as well as the primary tumor focus. Nevertheless, it is undeniable that with the progressive advancements in interventional therapies like HAIC and promising outcomes from clinical prospective trials, a multidisciplinary synergistic therapy centered around HAIC will offer an expanded array of therapeutic possibilities for liver metastases, ultimately leading to more definitive therapeutic effects.
